# Relationship between Microcirculatory Perfusion and Arterial Elastance: A Pilot Study

**DOI:** 10.1155/2019/3256313

**Published:** 2019-03-26

**Authors:** Ottavia Bond, Paolo De Santis, Enrica Iesu, Federico Franchi, Jean-Louis Vincent, Jacques Creteur, Fabio Silvio Taccone, Sabino Scolletta

**Affiliations:** ^1^Department of Intensive Care, Erasme Hospital, Université Libre de Bruxelles, Route de Lennik, 808, 1070 Brussels, Belgium; ^2^Department of Medicine, Surgery and Neuroscience, Anesthesia and Intensive Care, Siena University Hospital, Viale Bracci 11, 53100 Siena, Italy

## Abstract

**Background:**

Arterial elastance (Ea) represents the total afterload imposed on the left ventricle, and it is largely influenced by systemic vascular resistance (SVR). Although one can expect that Ea is influenced by peripheral endothelial function, no data are available to support it in patients. The aim of this study was to investigate the relationship between Ea, SVR, and microvascular perfusion in critically ill patients undergoing the fluid challenge (FC).

**Methods:**

A prospective study in patients receiving a fluid challenge. A pulse wave analysis system (MostCare, Vygon, France) was used to estimate Ea and an incident dark field (IDF) handheld device (Braedius Medical BV, The Netherlands) to evaluate the sublingual microcirculation. Microvascular perfusion was assessed using the proportion of small-perfused vessels (PPV). Relative changes in each variable were calculated before and after FC; fluid responsiveness was defined as an increase in the cardiac index by at least 10% from baseline.

**Results:**

We studied 20 patients requiring a fluid challenge (*n*=10 for hypotension; *n*=5 for oliguria; *n*=3 for lactate values greater than 2 mmol/l; *n*=2 for tachycardia), including 12 fluid responders. There was a strong correlation between Ea and SVR (*r*^2^ = 0.75; *p* < 0.001) and only a weak correlation between Ea and PPV at baseline (*r*^2^ = 0.22; *p*=0.04). Ea decreased from 1.4 [1.2–1.6] to 1.2 [1.1–1.4] mmHg/mL (*p*=0.01), SVR from 1207 [1006–1373] to 1073 [997–1202] dyn ∗ s/cm^5^ (*p*=0.06), and PPV from 56 [51–64] % to 59 [47–73] % (*p*=0.25) after fluid challenge. Changes in Ea were significantly correlated with changes in SVR, but not with changes in PPV.

**Conclusions:**

The correlation between Ea and indexes of microvascular perfusion in the sublingual region is weak. The impact of microcirculatory perfusion on the arterial load is probably limited.

## 1. Introduction

Cardiovascular alterations in the critically ill, which are frequently characterized by arterial hypotension, require prompt and adequate correction to prevent organ failure [1, 2]. Arterial elastance (Ea), a surrogate of arterial afterload usually calculated as the ratio of left ventricular end-systolic pressure and stroke volume, is an important determinant of arterial blood pressure; it reflects the net arterial load on the left ventricular ejection, and it is influenced by systemic vascular resistance (SVR) [3]. It is therefore expected to be correlated with the peripheral endothelial function (e.g., microcirculatory blood flow) [4].

Fluid therapy is one of the most commonly used therapeutic intervention, which aims at correcting hypovolemia, increasing cardiac preload and cardiac output, and potentially improving microcirculatory blood flow [2, 5, 6]. Fluid therapy may thus influence either Ea or the microvascular function, i.e., the number of perfused capillaries and the proportion of perfused small vessels (PPV) in this setting [5–7]. The integrity of endothelium, which is one of the major determinants of microcirculatory flow, significantly influences the vascular tone [4]. In addition, the microvascular local control of the flow leads to capillary dilatation or constriction on the basis of the local oxygen saturation. The increase of microvascular perfusion following fluid administration seems to be due to an increase of the driving pressure or a decrease of blood viscosity. However, fluid infusion can lead to microcirculatory oxygen saturation improvement and nitric oxide release which, in turn, is responsible for capillary dilatation and changes in vascular tone [4]. And so, one could hypothesize a potential association between Ea and microvascular perfusion. Some studies have shown that fluid administration improves microvascular perfusion, with an increased proportion of perfused capillaries or microvascular flow [5, 6]. These changes were independent of the effects on macrohemodynamic parameters (i.e., CO and arterial pressure) and were observed in particular in the first days of therapy [5, 6]. However, whether the effects of fluid therapy are similar on Ea and PPV remains unknown.

The aim of this study was to investigate the relationship between Ea, SVR, and PPV in critically ill patients who received a fluid challenge in an attempt to correct signs of tissue hypoperfusion.

## 2. Methods

This is a retrospective analysis of prospectively collected data conducted in a 35-bed mixed Department of Intensive Care between January and May 2018. The study was approved by the ethical committee (P2018/128), which waived the need for an informed consent, considering the retrospective nature of the study. We studied adult (>18 years old) patients, treated by mechanical ventilation, who received a fluid challenge (FC), while they were equipped with a radial arterial catheter for routine arterial pressure (AP) and cardiac output monitoring using an uncalibrated pulse-contour method device. Exclusion criteria were as follows: diseases that could affect the quality and reliability of the arterial signal, such as aortic valve disease, aortic aneurysms, and cardiac arrhythmias (atrial fibrillation or multiple ectopic beats), and poor quality of the arterial pressure signal due to excessive over- or underdamping of the catheter-transducer system checked using the fast flush test.

A fluid challenge was performed because of clinical or biological signs of impaired organ perfusion and according to the clinical judgment of the attending physician based on hemodynamic monitoring variables, as indicated by one or more of the following: tachycardia (>100 beats/min); mean arterial blood pressure (MAP) lower than 65 mmHg; oliguria (<0.5 ml/kg/h); blood lactate level greater than 2 mEq/L.

Hemodynamic and microcirculatory indices were recorded in a patient data management system (PDMS, Picis Critical Care Manager, Picis Inc., Wakefield, USA). Hemodynamic stability was required, as defined by a MAP varying by less than 10% during the assessment of the sublingual microcirculation. The ventilator settings, sedative, and vasoactive drugs infusion rates were kept constant throughout the evaluation. Tidal volume was set at 6–8 ml/Kg, with a respiratory rate to maintain the PaCO_2_ between 35 and 45 mmHg. FC was performed over 20 minutes using a minimal volume of 500 mL of crystalloid or colloid solutions. Unfortunately, there is no agreement on which specific increase of CO after FC should be considered to define a patient as “responder” [8]. In our study, an increase of 10% of the cardiac output (CO) after the fluid challenge was considered [9].

Hemodynamic and microcirculatory parameters were then assessed at the baseline (T_0_, i.e., before the fluid challenge) and then at the end of the fluid challenge (T_1_).

### 2.1. Hemodynamic and Arterial Elastance Assessment

Hemodynamic monitoring was achieved using MostCare^up^ (Vygon, Ecouen, France), an uncalibrated pulse-contour method (PCM) [10] connected to the radial arterial line with a standard transducer (Edwards Lifesciences, Irvine, CA). Before data acquisition and after zeroing at the atrial level, the arterial waveform signal was checked for quality [8]. Before and after fluid challenge, a full set of hemodynamic data was obtained, including heart rate (HR), MAP, and CO. Arterial elastance (Ea) was calculated as dicrotic pressure/SV (normal values: 1.1–1.4 mmHg/ml). Systemic vascular resistance (SVR) was calculated according to standard formulas. Blood lactate concentrations were also recorded.

### 2.2. Microcirculatory Measurements and Analysis

Microcirculation measurements were also obtained before and after the fluid challenge, and a third-generation incident dark field (IDF) microscope (CytoCam; Braedius Medical, Huizen, The Netherlands) was used [11, 12], which consists of a handheld computer-controlled device with a high-resolution image sensor. CytoCam-IDF shows 30% more capillaries, and it has a faster measurement acquisition time (3–5 seconds) than the previous devices [11, 12]. Without applying pressure, the tip of the light guide was gently placed on the mucosal surface of the sublingual area. A 10 second video recording of predefined (left, right, and midline sublingual cavity) sites was obtained in each patient. Recordings were then blinded and analyzed offline to obtain PPV, total vessel density, (TVD) and the perfused small vessel density (PVD), according to recent recommendations [13]. For each variable, the average of five consecutive measures was used for the analysis.

### 2.3. Statistical Analysis

Data were expressed as mean ± standard deviation (SD) or median (25^th^ to 75^th^ percentiles). The Kolmogorov–Smirnov test was used to verify the normality of distribution of continuous variables. The paired *t*-test, or the Wilcoxon test when appropriate, was used to compare the variables. To test the relationship between arterial elastance, microcirculatory perfusion, and systemic vascular resistance, Pearson's (or Spearman) correlation analysis was performed between Ea and PPV% and between Ea and SVR. Correlation coefficient and their 95% confidence interval were calculated. The percentage of variation (Δ) of the Ea, PPV, and SVR between T_0_ and T_1_ was calculated to assess the concordance between the variables changes. Statistical analysis was performed using GraphPad PRISM version 5.0 (San Diego, CA, USA). For all statistical tests, a *p* < 0.05 was considered as statistically significant.

## 3. Results

Twenty patients (median age 63 [53–71] years; male gender, *n*=14, 70%) were enrolled over the study period (Table 1). Most of them (*n*=18, 90%) received crystalloids. The median time from ICU admission to FC was 2 [1–2] days.

Overall, Ea decreased significantly from 1.4 [1.2–1.6] to 1.2 [1.1–1.4] mmHg/mL (*p*=0.01), and CVP increased from 7 [7–11] to 10 [8–12] (*p* < 0.01) after the fluid challenge, while SVR, PPV, MAP, and CO did not significantly change. Twelve patients (60%) were responders to fluid challenge (Table 2). In these patients, Ea decreased from 1.4 [1.3–1.7] to 1.2 [1.1–1.5] mmHg/mL (*p*=0.05) and SVR from 1266 [1082–1751] to 1073 [1002–1185] dyn ∗ s/cm^5^, (*p*=0.03) after FC. CVP increased from 7 [6–7] to 9 [7–10] mmHg (*p* < 0.01) and CO from 4.4 [3.4–5.0] to 5.0 [4.6–5.5] L/min (*p*=0.05) after FC. PPV and MAP did not change significantly. In the nonresponders, MAP increased significantly after FC (from 69 [65–71] to 73 [71–74] mmHg, *p*=0.01), while Ea, SVR, PPV, CVP, and CO did not significantly change. In particular, MAP increased in seven nonresponders. Five of these patients had a history of cardiomyopathy, one had a sepsis at the time of FC, and the last one was treated with V-V ECMO.

**Figure 1:**
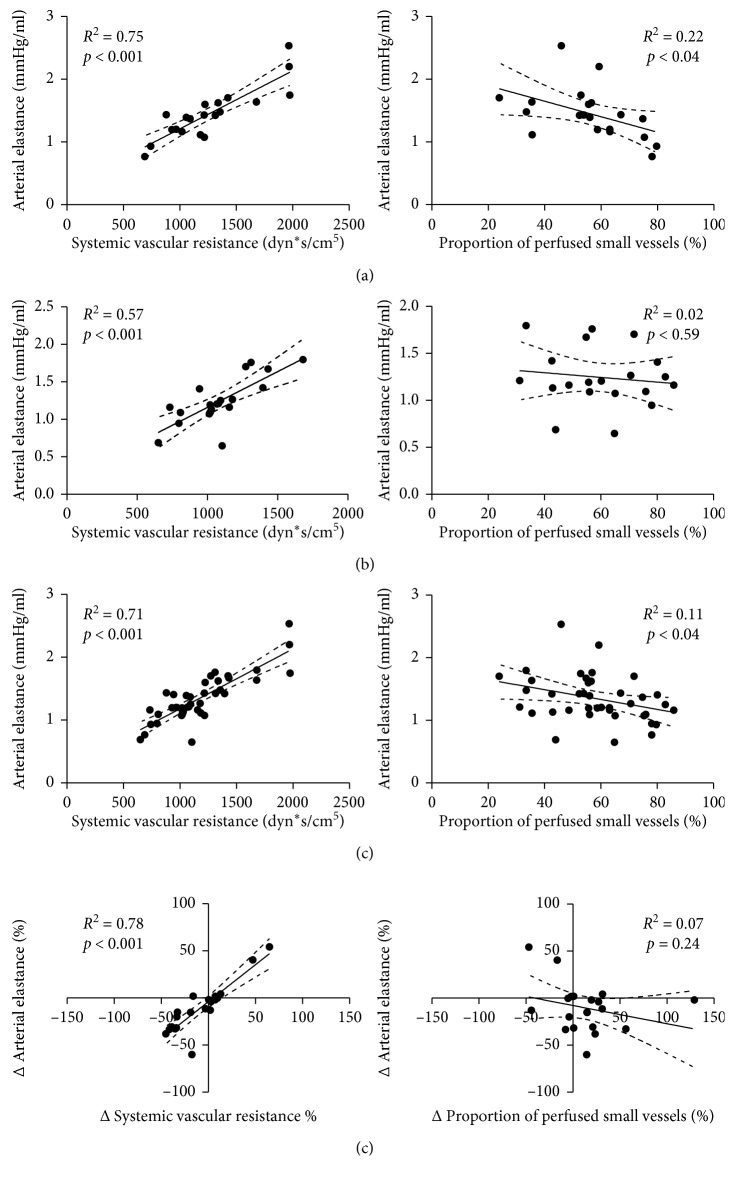
Correlation between arterial elastance and systemic vascular resistance (left column) and between arterial elastance and proportion of perfused small vessels (right column). (a) Baseline (T_0_); (b) after fluid challenge (T_1_); (c) all data (baseline plus after fluid challenge); (d) percentage of change between baseline and after fluid challenge.

**Figure 2:**
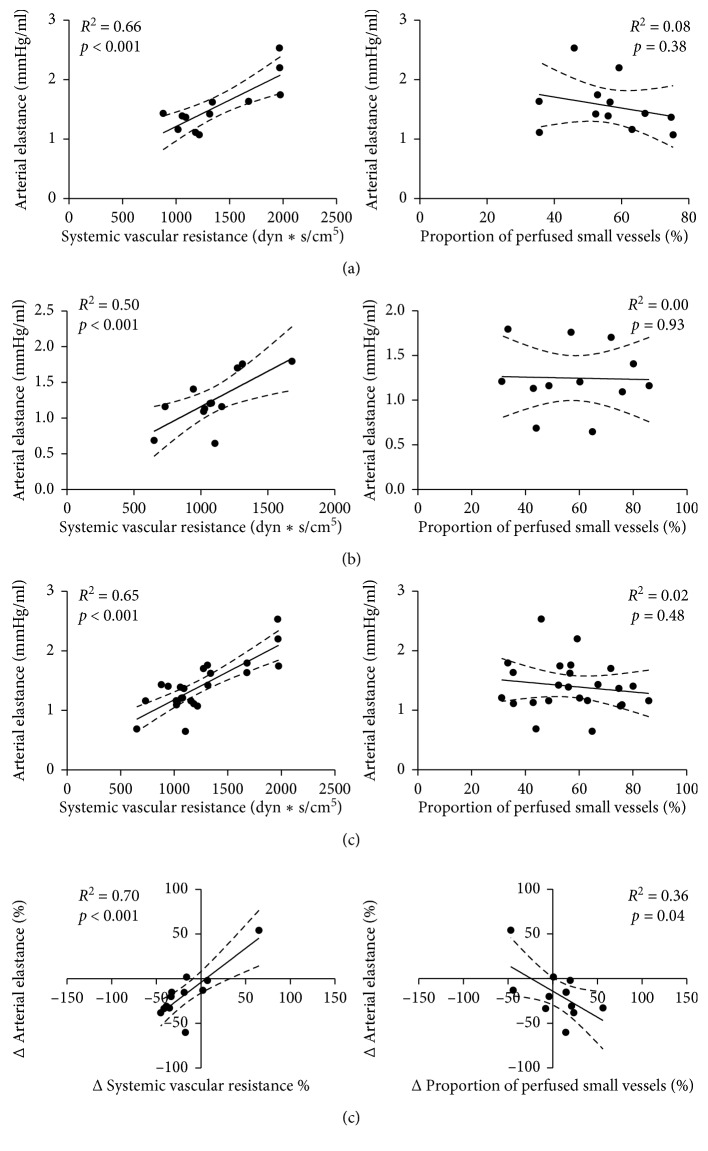
Correlation between arterial elastance and systemic vascular resistance (left column) and between arterial elastance and proportion of perfused small vessels (right column) in responders. (a) Baseline (T_0_); (b) after fluid challenge (T_1_); (c) all data (baseline plus after fluid challenge); (d) percentage of change between baseline and after fluid challenge.

**Figure 3:**
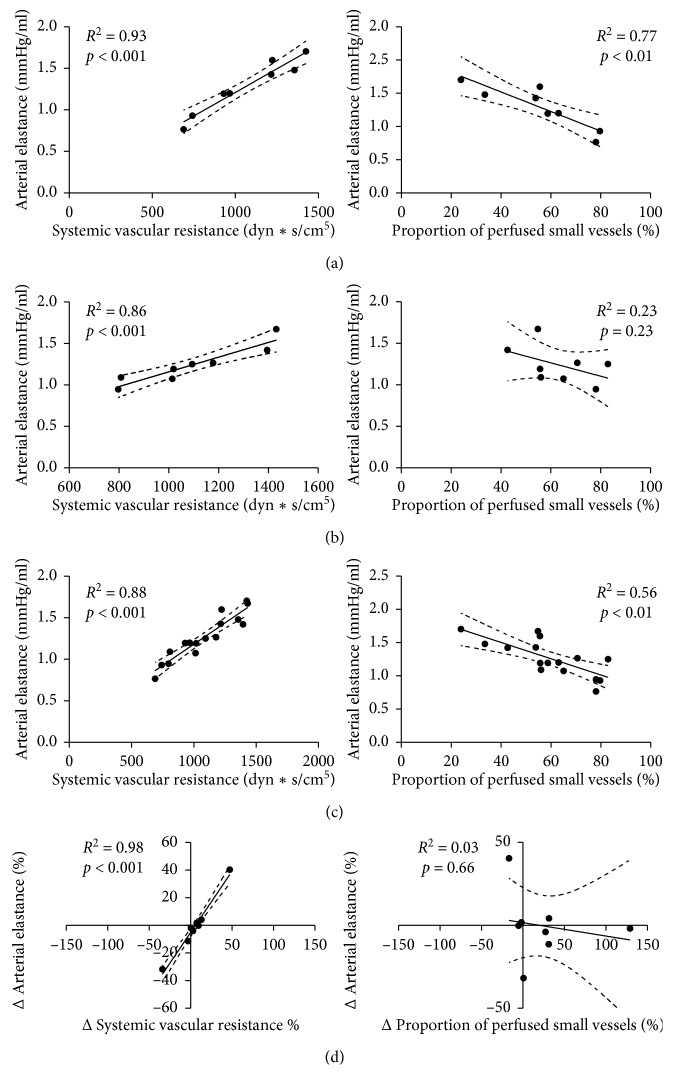
Correlation between arterial elastance and systemic vascular resistance (left column) and between arterial elastance and proportion of perfused small vessels (right column) in nonresponders. (a) Baseline (T_0_); (b) after fluid challenge (T_1_); (c) all data (baseline plus after fluid challenge); (d) percentage of change between baseline and after fluid challenge.

Overall, a significant correlation between Ea and SVR at T_0_, T_1_, and for all data (T_0_ + T_1_) was observed. A weak correlation between Ea and PPV was found at T_0_ and for all data, but not at T_1_. ΔEa was significantly correlated with ΔSVR but not with ΔPPV (Figure 1). Similar correlations were observed between Ea and SVR in responders and nonresponders and between Ea and PPV in nonresponders (Figures 2 and 3). There was no significant correlation between Ea and PPV in the responders.

## 4. Discussion

In the present study, we demonstrated a significant reduction of arterial elastance after the fluid challenge and a significant correlation between arterial elastance and systemic vascular resistance before and after fluid administration in critically ill patients. In contrast, we found only a weak correlation between arterial elastance and microvascular density in the sublingual area.

The fluid challenge is one of the most frequently used therapeutic interventions to increase arterial pressure and cardiac output in the presence of suspected hypovolemia. In responders, the fluid challenge induced a significantly increase in the cardiac output, while MAP did not change significantly. Physiologically, MAP is regulated by the sympathetic system, which tends to keep blood pressure constant, while the cardiac output might fluctuate [14]. In addition, for the same increase in the cardiac output, arterial blood pressure will change depending on the baseline arterial tone. In the presence of severe vasodilation, as it occurs in distributive shock, arterial pressure may not increase after a fluid challenge, even when the cardiac index does [14]. In particular, in septic patients, a ventriculoarterial decoupling may occur and could be associated with impaired left ventricular performance [15]. In this circumstance, the strategy to obtain an increase in CO could be represented by therapy aimed at maximizing the ventriculoarterial coupling, rather than increasing the circulating volume with fluid administration [15]. In the present study, five of seven nonresponders had a history of cardiopathy, which might justify a ventriculoarterial decoupling [15]. In addition, our study group has previously demonstrated that changes in arterial pressure could not reliably track changes in CO after fluid challenge [9]. Therefore, the degree of increase in arterial pressure is determined by both left ventricular ejection and arterial elastance. Arterial elastance represents the total afterload imposed on the left ventricle and is an expression of the complex interaction between different arterial properties, including compliance, wall stiffness, and outflow resistance [3]. As a consequence, the correlation between arterial elastance and systemic vascular resistance that we observed was physiologically expected. The decrease in arterial elastance after the fluid challenge was consistent with other studies and supported the value of arterial elastance to assess fluid responsiveness in critically ill patients [7, 16].

In the nonresponders, central venous pressure did not change significantly after the fluid challenge. The capacity of the vascular system to increase the mean arterial pressure may change during a fluid challenge [9]. Hence, fluid challenge would have not induced an increase in the cardiac output in nonresponders probably either due to a small amount of fluid administered or to an overdilated venous compartment (i.e., increased venous compliance). Moreover, in both these cases, the central venous pressure may not increase [17]. In fact, CVP represents only one of the determinants of venous return. The venous compliance and resistance and the stressed volume represent the other one. When the venous return curve intercepts Starling's curve in the preload independent part, a volume infusion will not increase stroke volume but will increase CVP. On the other hand, when the heart is working on the flat part of the venous return curve, CO will increase only increasing the stressed volume or decreasing venous resistance [18]. Moreover, based on Guyton's model, a low CO could be due to a decrease in preload (i.e., reduced venous return) or an impaired cardiac function (i.e., reduced myocardial contractility). High CVP values mean that, probably, a low CO is due to a decreased cardiac function, while low CVP values indicate a low preload. However, Guyton's model of the interaction between venous return and cardiac function clearly showed that single measurements of CVP are not representative of preload or cardiac performance. In fact, usually, healthy individuals have low CVP but normal CO values. Alike, low CVP can be observed during the loss of circulating volume and low cardiac output, independently from the cardiac function [18]. However, a positive response to fluids occurs more often when central venous pressure values are low, i.e., less than 6 mmHg, but it is less likely when values were greater than 15 mmHg [19]. This could, in part, justify the absence of changes in central venous pressure in our nonresponders, as they had intermediate values, while responders had lower baseline central venous pressure values. Hence, the presence of extreme central venous pressure values is more helpful to guide fluid administration than in case of values in the middle range [17].

In the present study, PPV did not significantly change after fluid challenge. As the previous studies showed a significant increase in PPV after fluid therapy in septic patients [5, 6], this apparently divergent results may be due to different patient populations. Indeed, in septic patients, microcirculation disturbances are often characterized by a decrease in capillary density in association with increased heterogeneous perfusion in visualized capillaries [4], while these changes may not be observed in other pathological conditions. Another potential explanation is that the identification of the fluid responder by an increase in CI may not directly translate into a clinically relevant response of the microvascular circulation, although we do not have enough patients in this cohort to assess this hypothesis. Also, a significant increase in the perfused capillary density in nonresponder patients was observed, which would require more patients to understand its significance.

We found only a weak correlation between PPV and arterial elastance. Similarly, other studies showed a weak, and often transitory, correlation between PPV and macrohemodynamic parameters (e.g., cardiac output, oxygen delivery, and venous oxygen saturation) in septic patients [20, 21]. It could be also speculated that arterial elastance may be more influenced by the arteriolar tone, while other factors, such as tissue edema, neural perivascular activity, and changes in red blood cells deformability, may impact on capillary density and perfusion. Also, it is necessary to recognize that Ea, as surrogate of afterload, is one of the determinants of arterial pressure, but it is not a direct variable affecting microcirculation. Arterial pressure is the driving pressure for microcirculatory perfusion, but microcirculatory pressure is usually independent of the arterial pressure [6]. However, when the autoregulatory mechanisms are lost, this relationship is established. On the other hand, the fluid challenge could lead to microcirculatory changes (e.g., improvement of the proportion of small-perfused vessels), which could be only a local and not systemic result [6]. Actually, any specific relationship between macro- and microcirculatory blood flow remains not completely understood.

Finally, we studied the microcirculation network in a specific organ (i.e., sublingual), while Ea assessed by the MostCare is the reflection of the vascular tone of the cannulated artery (i.e., radial artery), which would have a different response to therapeutic interventions and a different microvascular flow than the sublingual area. Our findings also suggest that potential surrogates of sublingual microvascular perfusion, such as SVR, cannot estimate microvascular function at the bedside and that handheld computer-controlled devices with a high-resolution image sensor are the only adequate tool to quantify capillary perfusion.

This study had some limitations. First, the relatively small number of patients enrolled limited the analysis between responders and nonresponders to fluid challenge; however, the aim of this study was to compare Ea to microcirculatory parameters before and after fluid challenge, and this association would remain weak even by adding more patients. Second, the majority of our patients received a crystalloid solution, and colloids or red blood cells transfusion may have different effects on the microcirculation. Also, the patient population was still heterogeneous, with a potentially different basal microcirculatory perfusion condition and different response to fluid therapy. Moreover, we were not able to stratify the severity of the patients at the time of fluid challenge and the evolution of the diseases after fluid administration, and we could not fully understand the effect of the fluid on microcirculation. We cannot exclude that the results of the present study could be only the consequence of the population studied and the timing on the evolution of the disease. Third, we assessed Ea using an uncalibrated pulse-contour waveform analysis device, while the “gold standard” is still considered the ventricular pressure-volume with invasive aortic pressure [22–24]. In addition, we estimated Ea as the ratio between dicrotic pressure and stroke volume. Dicrotic pressure could be greatly affected by viscoelastic arterial properties and arterial wave reflections [25], and we cannot exclude that these factors may have affected the pressure wave-derived measurements and biased our results. Fourth, we compared Ea with PPV, which are surrogate of arterial load and microcirculatory perfusion, respectively. As a consequence, we could not fully understand the exact relationship existing between arterial load and microcirculation. Fifth, the microvascular perfusion was investigated in a specific organ (i.e., sublingual), while arterial elastance was assessed at the level of the radial artery (related to a different microvascular networks than the sublingual area). Nevertheless, Ea and PPV changed differently after FC because of the “dissociation” between macro- and microhemodynamics in critically ill patients. As such, these two variables are not correlated, and this would be independent from the method used to assess Ea. Finally, we did not assess the dynamic elastance (Ea_dyn_), which could potentially be associated with changes in volume load and arterial tone more than the static one we measured.

## 5. Conclusions

Arterial elastance significantly decreases after the fluid challenge, and this change is correlated with systemic vascular resistance. We observed only a weak correlation between arterial elastance and microvascular perfusion, implying that the impact of microcirculatory perfusion on the arterial load is probably limited.

## Figures and Tables

**Table 1:** Characteristics of the study cohort.

	*N*=20
Age (years)	64 [53–71]
Weight (kg)	84 [74–90]
Male gender, *n* (%)	14 (70)
Chronic heart failure, *n* (%)	10 (50)
Hypertension, *n* (%)	12 (60)
Diabetes, *n* (%)	6 (30)
COPD/asthma, *n* (%)	3 (15)
Neurological disease, *n* (%)	6 (30)
Chronic renal disease, *n* (%)	2 (10)
Liver cirrhosis, *n* (%)	6 (30)
Immunosuppressive agents, *n* (%)	1 (5)
Reasons for ICU admission	
Cardiac arrest	4 (20)
After heart surgery	4 (20)
Acute brain injury	4 (20)
Hemorrhagic shock	3 (15)
Respiratory failure	3 (15)
Liver transplantation	1 (10)
Lung transplantation	1 (10)
SOFA score on the day of FC	6 [5–8]
Vasopressor therapy, *n* (%)	7 (35)
Inotropic therapy, *n* (%)	2 (10)
Mechanical ventilation, *n* (%)	8 (40)
CRRT therapy, *n* (%)	2 (10)
Reasons for fluid challenge	
Oliguria	10 (50)
Mean arterial blood pressure < 65 mmHg	5 (25)
Lactate values > 2 mmol/L	3 (15)
Tachycardia	2 (10)
ICU stay	4 [3–9]
ICU mortality, *n* (%)	5 (25)

COPD = chronic obstructive pulmonary disease; ICU = intensive care unit; SOFA = sequential organ failure assessment; CRRT = continuous renal replacement therapy; FC = fluid challenge.

**Table 2:** Hemodynamic and microvascular perfusion parameters, with regard to responsiveness to fluid challenge (FC).

Variable	All patients (*n*=20)		Responders (*n*=12)		Nonresponders (*n*=8)	
	Baseline	After FC	Baseline	After FC	Baseline	After FC
Global hemodynamics						
MAP (mmHg)	71 [68–81]	74 [71–77]	79 [69–88]	74 [72–84]	69 [65–71]	73 [71–74]^*∗*^
CVP (mmHg)	7 [7–11]	10 [8–12]^*∗*^	7 [6–7]	9 [7–10]^*∗*^	10 [9–12]	10 [10–12]
HR (bpm)	94 [81–102]	90 [80–103]	94 [82–102]	93 [80–104]	90 [81–100]	87 [80–94]
CO (L/min)	4.3 [3.5–5.0]	4.9 [4.2–5.4]	4.4 [3.4–5.0]	5.0 [4.6–5.5]^*∗*^	4.0 [3.5–5.2]	4.8 [3.9–5.0]
Ea (mmHg/ml)	1.4 [1.2–1.6]	1.2 [1.1–1.4]^*∗*^	1.4 [1.3–1.7]	1.2 [1.1–1.5]^*∗*^	1.3 [1.1–1.5]	1.2 [1.1–1.3]
SVR (dyn ∗ s/cm^5^)	1207 [1006–1373]	1073 [997–1202]	1266 [1082–1751]	1073 [1002–1185]^*∗*^	1091 [883–1256]	1056 [963–1233]
Lactate, mmol/L	1.7 [0.9–2.2]	1.7 [0.9–2.1]	1.6 [1.0–2.0]	1.7 [0.9–2.1]	1.7 [0.9–2.9]	1.7 [0.9–2.3]

Microcirculation						
PPV (%)	56 [51–64]	59 [47–73]	56 [51–64]	58 [44–73]	57 [49–67]	60 [55–72]
TVD (mm/mm^2^)	17 [14–19]	17 [16–20]	18 [15–21]	18 [16–21]	15 [13–16]	15 [13–16]
PVD (mm/mm^2^)	9 [7–11]	10 [8–12]	10 [8–12]	10 [7–13]	7 [5–9]	10 [9–12]^*∗*^

FC, fluid challenge; MAP, mean arterial pressure; HR, heart rate; CO, cardiac output; Ea, arterial elastance; SVR, systemic vascular resistances; PPV, proportion of perfused small vessels; TVD, total vessel density; PVD, perfused small vessel density, ^*∗*^*p* < 0.05.

## Data Availability

The data used to support the findings of this study are available from the corresponding author upon request.
